# Effect of *FTO* rs9939609 variant on insulin resistance in obese female adolescents

**DOI:** 10.1186/s13104-018-3392-8

**Published:** 2018-05-15

**Authors:** Kristy Iskandar, Suryono Yudha Patria, Emy Huriyati, Harry Freitag Luglio, Madarina Julia, Rina Susilowati

**Affiliations:** 1 Department of Child Health, Faculty of Medicine, Universitas Gadjah Mada/UGM Academic Hospital, Jl. Kabupaten (Lingkar Utara), Kronggahan, Trihanggo, Gamping, Sleman, Yogyakarta, 55291 Indonesia; 2 Department of Child Health, Faculty of Medicine, Public Health and Nursing, Universitas Gadjah Mada/Dr. Sardjito Hospital, Yogyakarta, Indonesia; 3 Department of Nutrition and Health, Faculty of Medicine, Universitas Gadjah Mada, Yogyakarta, Indonesia; 4 Department of Histology and Cell Biology, Faculty of Medicine, Universitas Gadjah Mada, Yogyakarta, Indonesia

**Keywords:** Common variant, *FTO*, rs9939609, Indonesia, Insulin resistance, Obese female adolescents

## Abstract

**Objectives:**

*FTO* rs9939609 variant has been shown to be associated with insulin resistance in Caucasian children. However, studies in Asia show inconsistent findings. We investigated the association between *FTO* rs9939609 polymorphisms and insulin resistance in obese female adolescents in Indonesia, a genetically distinct group within Asia.

**Results:**

A total of 78 obese female adolescents participated in this study. The risk allele (A) frequency of *FTO* rs9939609 variant in Indonesian obese female adolescence was 44.2%. The frequency of insulin resistance was higher in the subjects with AA (54.6%) or AT (59.6%) than the subject with TT genotype (50%), but did not statistically different (p = 0.81 and p = 0.47, respectively). The insulin resistance rate was also higher in the risk allele (A) than the non-risk allele (T) subjects (0.58 vs. 0.55), but did not statistically different (p = 0.75). There was no association between *FTO* rs9939609 variant and body mass index, fasting glucose level, fasting insulin level, homeostatic model assessment of insulin resistance, and waist circumference (p > 0.05). In conclusion, *FTO* rs9939609 variant may not be associated with insulin resistance in Indonesian obese female adolescents. A multicenter study with a larger sample size is needed to clarify these findings.

## Introduction

Obesity is one of the major health problems all over the world. The obesity incidence in children and adolescents has increased over the last two decades. Obesity is related to other diseases such as insulin resistance. Recently, the prevalence of obesity in Indonesian children and adolescents increased to 10% [1]. It is suggested that there are genetic factors that affect the insulin resistance in obesity.

*Fat mass and obesity related gene (FTO)*, located at 16q12.2, is associated with obesity and type 2 diabetes mellitus (T2DM). *FTO* rs9939609 common variant has been significantly related with increased insulin resistance in obese female adolescents (p < 0.001) [2], but not in male adolescents in Chile after adjustment for body mass index (BMI). However, other studies did not show any association between *FTO* rs9939609 polymorphism and insulin resistance [3, 4].

Indonesia is a country with approximately 400 ethnic and linguistic groups [5]. Indonesian native ancestries are genetically similar to Asians, with some genetic evidence of a division between East and Southeast Asians [6, 7]. There appears to be some differences in allele frequencies of common variants within Asian populations according to the Y chromosome polymorphism findings in North Asians, Han Chinese, Japanese and Southeast Asians [8]. Thus, Indonesian obese female adolescents can shed additional light on the *FTO* rs9939609 effect on insulin resistance by providing data from a genetically distinct group within Asia.

## Main text

### Methods

#### Subjects

We enlisted obese female adolescents aged 11–15 years old at six junior high schools in Yogyakarta, Indonesia. We determined their height and weight to calculate the BMI and categorize them into underweight, normal, overweight or obese. The prevalence of obesity among those female adolescents was 136/2101 (6.4%).

After getting informed consent, we collected the blood samples from 78 obese female adolescents. The inclusion criteria of this study were as follows: (1) female adolescent aged 11–15 years old; (2) obese (BMI ≥ 95%) according to the Center for Disease Control and Prevention criteria based on age and sex; (3) reached menarche; and (4) signed informed consent form, while the exclusion criteria consisted of: (1) incomplete data; and (2) poor quality of DNA. The study has been approved by Institutional Review Board of Faculty of Medicine Universitas Gadjah Mada/Dr. Sardjito Hospital (KE/FK/183/EC).

#### Insulin resistance measurement

Insulin resistance was determined according to homeostatic model assessment of insulin resistance (HOMA-IR), and calculated by the following formula: fasting glucose level (mg/dL) × fasting insulin level (mU/mL)/405. The subject with the HOMA-IR of ≥ 3.16 was classified as having insulin resistance. As for the measurement of fasting glucose and insulin level, blood sampling was taken in the morning after fasting 8–12 h (last meal at 10 pm). Blood insulin level was determined using the immunoassay method, whereas glucose level was measured by the hexokinase technique.

#### Genotyping

DNA was extracted from a 5-mL blood using salting out method. Genotyping of FTO rs9939609 was determined using polymerase chain reaction (PCR) twice. The PCR was conducted using GoTaq^®^PCR reaction mixture. The first PCR used an allele A specific primer, while the second PCR used an allele T specific primer. The PCR conditions were 94 °C for 3 min, followed by 35 cycles of 94 °C for 30 s, 65 °C for 30 s, then 72 °C for 30 s, and finally 72 °C for 5 min. To ensure the validity, the genotyping was determined by two independent experts. If any difference existed, the PCR was repeated.

#### Statistical analysis

The association between the *FTO* rs9939609 variants (TT, TA, AA) and insulin resistance was determined by Chi square test. Data distribution was tested by the Kolmogorov Smirnov test. If the data distribution was abnormal, the Mann–Whitney U and Kruskal–Wallis tests were used for continue and category data analysis. Logistic regression was applied to adjust for differences in BMI between insulin resistance and non-insulin resistance groups. The analysis was performed using SPSS ver. 20.0 for Windows (SPSS, Inc. Chicago, IL).

### Results

**Fig. 1:**
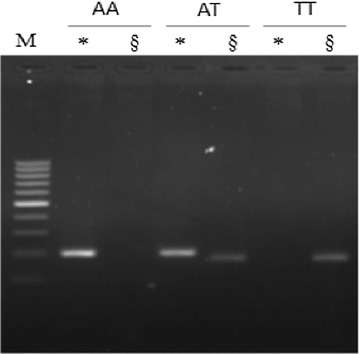
PCR products of FTO rs9939609. *Showed A allele specific PCR product (201 bp), while ^§^showed T allele specific PCR product (178 bp). We conducted two times PCR for each sample. Positive result o n A allele specific PCR product but negative on T allele specific PCR product means AA genotype (lanes 1 and 2). Both positive A and T allele specific PCR product means AT genotype (lanes 3 and 4). Negative result on A allele specific PCR product but positive on T allele specific PCR product means TT genotype (lanes 5 and 6)

There were three genotypes of *FTO* rs9939609 found in the subjects: AA, AT and TT (Fig. 1). The risk allele (A) frequency of *FTO* rs9939609 variant in Indonesian obese female adolescence was 44.2%. Among 78 obese female adolescents, 25.6% were TT, 60.2% were AT, and 14.1% were AA. The frequency of insulin resistance was higher in the subjects with AA (54.6%) or AT (59.6%) than the subject with TT genotype (50%), however, these differences did not reach a significant level (p = 0.81 and p = 0.47, respectively) (Table 1). The insulin resistance rate was also higher in the risk allele (A) than the non-risk allele (T) subjects (0.58 vs. 0.55, respectively), but did not statistically different (0.75) (Table 1).

**Table 1:** The frequency of genotype and allele of *FTO* rs9939609 polymorphism in insulin resistance (IR) and non-insulin resistance (NIR) groups

	N	%	NIR (%)	IR (%)	OR (95% IK)	p
Genotypes						
AA	20	25.6	5 (45.5)	6 (54.5)	1.2 (0.2–6.5)	0.81
AT	47	60.3	19 (40.4)	28 (59.6)	1.5 (0.5–9.8)	0.47
TT	11	14.1	10 (50)	10 (50)	Reference	
Alleles						
A	87	44.2	29 (42)	40 (58)	1.1 (0.6–2.1)	0.75
T	69	55.8	39 (44.8)	48 (55.2)	Reference	

*IR* insulin resistance, *NIR* non-insulin resistance

This study further aimed to evaluate the effect of *FTO* rs9939609 variants on the following parameters: BMI, fasting glucose level, fasting insulin level, HOMA-IR and waist circumference. All measurements were higher in the AA genotype group compared to other genotype groups, however the results did not reach significant levels (Table 2). Accordingly, the results showed that there was no significant association statistically between risk allele A rs9939609 variant and BMI, fasting glucose level, fasting insulin level, HOMA-IR and waist circumference (Table 2).

**Table 2:** The association between body mass index (BMI), fasting glucose level, fasting insulin level, HOMA-IR and waist circumference and *FTO* rs9939609 variant genotypes and alleles

Parameters	Genotypes			p	Alleles		p
	AA	AT	TT		A	T	
BMI^a^	30.79 ± 3.39	29.44 ± 2.37	29.61 ± 2.59	0.30	29.87 ± 2.76	29.52 ± 2.45	0.32
Fasting glucose^b^	89.73 ± 13.13	89.96 ± 21.51	83.5 ± 8.47	0.39	89.80 ± 19.07	86.99 ± 17.02	0.72
Fasting insulin^c^	19.82 ± 15.03	17.73 ± 11.11	16.55 ± 8.37	0.74	18.39 ± 12.28	17.19 ± 9.86	0.34
HOMA-IR	4.29 ± 3.43	4.11 ± 3.69	3.41 ± 1.75	0.68	4.17 ± 3.56	3.79 ± 2.96	0.37
Waist circumference^d^	91.59 ± 8.76	88.97 ± 5.94	88.25 ± 6.53	0.38	89.8 ± 6.92	86.99 ± 17.02	0.35

Data was presented with mean ± SD. *p* value was determined using Kruskal–Wallis test since the data distribution was abnormal according to Kolmogorov–Smirnov test (p < 0.05)

^a^kg/m^2^; ^b^ mg/dL; ^c^ µM/mL; ^d^ cm

To assess the effect of population stratification, we analyzed the total Indonesian sample genotypes (44 IR subjects and 34 NIR subjects) for the Hardy–Weinberg equilibrium: rs9939609 (p > 0.05) showed no departures from expectations.

### Discussion

In this study, we were unable to show any significant association between *FTO* rs9939609 common variant and insulin resistance. It should be noted that although not statistically significant, BMI, fasting glucose level, fasting insulin level, HOMA-IR and waist circumference are higher in individuals with risk allele (A) compared to those with non-risk allele (T).

Zavattari et al. [4] also showed that there was no association between *FTO* rs9939609 polymorphism and biochemistry parameters such as HOMA-IR, serum insulin levels and oral glucose tolerance test in obese adolescents in Italy. Shimaoka et al. [3] described the association between *FTO* rs1121980 variant (*not* rs9939609) and insulin resistance in a Japan population. However, this finding differed from other studies in Chile, Scotland and Finland [2, 9, 10].

In this study, the risk allele A frequency in obese female adolescents in Yogyakarta was 44.2%. This frequency was lower than the frequency in Western countries (55–65.4%) [2, 4], but higher than in a control population in Asia (16%), or world population (36%) that consisted of: Americans (28%), Europeans (42%), and Asians (16%), except Africans (54%) [11, 12].

There are several established findings regarding the association between insulin resistance and *FTO* rs9939609. Individuals with rs9939609 risk allele A have more *FTO* transcripts than individuals with allele T [13]. The mRNA *FTO* was associated with gene expression demonstrated to be important for glucose homeostasis [14], gene expression, which involved gluconeogenesis in liver [15], *TNF* and *NFKB1* level in subcutaneous lipid [16] and insulin and mRNA *KCNJ11* in beta cells, in which these genes were concluded to be responsible for glucose homeostasis. One study showed that overexpression of *FTO* in *INS*-*1* pancreatic beta cells increased the first phase of insulin secretion response to glucose [17].

Those mechanisms provide possible explanations about how the *FTO* polymorphisms influence the insulin resistance. Additionally, previous studies showed that rs9939609 variants increased the *FTO* mRNA transcript important for gene expression involved in glucose homeostasis and insulin resistance. Furthermore, *FTO* is also associated with nucleic acid demethylation and co-activator transcription that regulate the expression of other genes.

The discrepancy in findings concerning the association between *FTO* rs9939609 variants and insulin resistance among other studies including our results indicates that the effect of *FTO* rs9939609 variants on insulin resistance may be influenced by other variables including: gender, age and ethnic [18–21]. The differences in our results may also be due to the limitations created by the small sample population [2, 20, 21].

### Conclusions

*FTO* rs9939609 variant may not be associated with insulin resistance in Indonesian obese female adolescents. This emphasizes the necessity to perform a multicenter study with a larger sample size to clarify the impact of *FTO* rs9939609 variant on insulin resistance in obese female adolescent in Indonesia.

## Limitations

First, it is difficult to conclude the causality based on a case–control design; a follow-up longitudinal study is recommended to provide confirmation of our findings. In addition, this study only examined one polymorphism, *FTO* rs9939609, among 40 common variants that are connected to each other in current comparisons of linkage disequilibrium such as the genomic HapMap description of r^2^ > 0.80 in Caucasian populations that demonstrates intervariability among ethnic groups in the present Human Genome Project efforts to map the DNA of *Homo sapiens* [12].

## Abbreviation

FTO: fat mass and obesity related
